# Comparison of ultrasound-guided percutaneous radiofrequency ablation and reoperation for nerve-adjacent cervical lymph node recurrence of papillary thyroid carcinoma: a propensity score–matched study

**DOI:** 10.3389/fendo.2026.1852967

**Published:** 2026-06-11

**Authors:** Yuhan Xie, Yuhan Qiu, Lingpeng Tang, Ting Hu, Songsong Wu, Jianchuan Yang

**Affiliations:** 1 Shengli Clinical Medical College of Fujian Medical University, Fujian Medical University, Fuzhou, China; 2 Department of Ultrasonography, Fujian Provincial Hospital, Fuzhou University Affiliated Provincial Hospital, Fuzhou, China

**Keywords:** cervical lymph node metastasis, papillary thyroid carcinoma, perineural regions, reoperation, ultrasound-guided radiofrequency ablation

## Abstract

**Objectives:**

To compare the short-term efficacy and safety of ultrasound-guided percutaneous radiofrequency ablation (RFA) and reoperation (RO) for recurrent papillary thyroid carcinoma presenting as solitary cervical lymph node metastasis (R-PTC-SCLNM) located in perineural regions (PNRs).

**Methods:**

This retrospective study included patients with R-PTC-SCLNM in PNRs who underwent RFA or RO. Propensity score matching was performed to balance baseline characteristics. Kaplan–Meier analysis was used to assess recurrence-free survival, and treatment outcomes and complications were compared between groups. Subgroup analyses were conducted according to the anatomical relationship between metastatic lymph nodes and adjacent nerves.

**Results:**

After PSM, 71 matched pairs were included. In the RFA group, the mean volume reduction ratio (VRR) at 24 months was 0.97 ± 0.04, and 42.3% of metastatic lymph nodes had completely disappeared. The overall local recurrence rate did not differ significantly between the RFA and RO groups, consistent with the Kaplan–Meier analysis. The RFA group had significantly lower overall complication rates than the RO group (P<0.001), including both nerve-related complications (P = 0.009) and non-nerve-related complications (P = 0.002). Subgroup analysis showed that this safety difference was most evident in Type II lesions. The pre-PSM sensitivity analysis showed a similar trend. In addition, compared with RO, RFA was associated with significantly shorter procedure time, shorter hospital stay, less intraoperative blood loss, and smaller postoperative scar length (all P<0.001).

**Conclusion:**

In carefully selected patients with nerve-adjacent R-PTC-SCLNM, RFA demonstrated acceptable short-term local control and a minimally invasive profile characterized by faster recovery and a lower complication burden. RFA may serve as a potential treatment option for patients who are unsuitable for or unwilling to undergo reoperation.

## 1 Introduction

The most common metastatic pathway of papillary thyroid carcinoma (PTC) is regional lymph node metastasis (LNM) (1). Despite the use of standardized strategies involving thyroidectomy combined with central and/or lateral neck lymph node dissection during the initial surgery (2), approximately 5%–20% of patients still experience local recurrence or newly developed LNMs during follow-up (3), making the selection of treatment for recurrent nodal metastasis a common yet highly challenging clinical problem in thyroid cancer management (4). Previous studies have shown that most recurrent lymph nodes appear within 2–10 years after surgery (5), predominantly involving the central compartment (level VI) and lateral neck compartments (levels II–V) (6). Among these, some recurrent lymph nodes are located in anatomically complex regions associated with a high functional risk (7), particularly the perineural regions (PNRs), which not only increase the technical difficulty of surgical procedures but also may increase the risk of intraoperative nerve injury and postoperative functional impairment, thereby posing greater challenges for clinical treatment.

PNRs refer to high-risk anatomical regions located in close proximity to critical neural structures and requiring special attention during surgical procedures. Lymph nodes in these regions are primarily distributed around the recurrent laryngeal nerve (RLN), vagus nerve (VN), cervical sympathetic trunk (CST), and accessory nerve (AN), and are located across the central compartment (level VI), lateral neck compartments (levels II–V), and deep cervical fascial spaces (8). Typical anatomical locations include the paratracheal space and regions adjacent to the carotid sheath. Given that these nerves course within the deep cervical fascia and may form complex spatial relationships with metastatic lymph nodes, this anatomical proximity may increase the technical difficulty of lymph node dissection and the risk of nerve injury even in the absence of definite neural invasion (9). Therefore, PNRs represent not only high-risk anatomical sites for nodal recurrence of PTC but also critical regions influencing treatment safety and functional preservation.

Reoperation with cervical lymph node dissection remains the primary treatment strategy for recurrent papillary thyroid carcinoma with solitary cervical lymph node metastasis (R-PTC-SCLNM) (2). However, prior surgery may make cervical neurovascular structures more vulnerable, and postoperative scarring and fibrosis can disrupt normal anatomical planes, thereby markedly increasing the complexity of reoperation (10). Particularly within PNRs, re-exposure and dissection of tissue planes are more challenging, and even minor deviations in surgical manipulation may lead to severe complications, such as permanent RLN injury and Horner’s syndrome, with adverse effects on patients’ quality of life and long-term functional recovery (11).

In recent years, ultrasound-guided radiofrequency ablation (RFA) has gained increasing attention as a minimally invasive treatment for R-PTC-SCLNM, owing to its potential advantages, including low invasiveness, real-time visualization, and faster recovery (12). Compared with open surgery, RFA avoids extensive dissection of scar tissue in previously operated areas; under real-time ultrasound guidance, it enables precise needle placement and uses hydrodissection to establish a thermal protective barrier, thereby inducing coagulative necrosis and local inactivation of the target lesion (13). Therefore, RFA may provide a low-trauma treatment option for carefully selected patients in whom the lesion is clearly visualized and safe hydrodissection is feasible. However, compared with RO, the efficacy and safety of RFA for nerve-adjacent R-PTC-SCLNM have not been systematically evaluated.

This study compared short-term local control, perioperative recovery, and treatment-related complications between RFA and RO in patients with nerve-adjacent R-PTC-SCLNM, aiming to provide evidence to guide treatment selection for R-PTC-SCLNM in PNRs.

## 2 Materials and methods

### 2.1 Patients

This study was approved by the Institutional Review Board of Fujian Provincial Hospital (K2026-03-024). Given the retrospective design, the requirement for informed consent for patient data disclosure was waived. In addition, all patients provided written informed consent before undergoing RFA or RO.

We retrospectively reviewed the clinical data of patients with R-PTC-SCLNM located in cervical PNRs who were treated at Fujian Provincial Hospital between January 2018 and December 2022, including medical records, ultrasound imaging findings, fine-needle aspiration (FNA) or core-needle biopsy (CNB) results, surgical records, and relevant laboratory tests. All patients completed a uniform 24-month follow-up. In clinical practice, RO remains an important treatment option for R-PTC-SCLNM, and I-131 therapy may be applied to lesions with radioactive iodine uptake. For patients who were unsuitable for or refused RO, and whose lesions were localized, clearly visualized on ultrasound, and amenable to safe hydrodissection, RFA could be considered as a local minimally invasive treatment option.

The inclusion criteria were as follows: (1) prior thyroidectomy with exactly one previous cervical lymph node dissection; (2) suspicious recurrent cervical lymph nodes confirmed as metastatic PTC by FNA or CNB; (3) metastatic lymph nodes located in PNRs, defined as lesions adjacent to or in contact with critical neural structures, including the RLN, VN, CST, or AN, on imaging or preoperative assessment, with a high surgical risk of nerve injury; (4) a single pathologically confirmed recurrent lymph node with a maximum diameter ≤30 mm; and (5) no history of cervical radiotherapy. The exclusion criteria were as follows: (1) age <16 years or pregnancy; (2) imaging suspicion without histological confirmation; (3) distant metastasis; and (4) inability to comply with treatment or follow-up, or refusal to provide informed consent.

### 2.2 Pre-treatment assessment

All patients underwent pretreatment assessment with ultrasound, ultrasound-guided FNA or CNB, and laboratory testing. Conventional ultrasound documented lymph node size including three diameters, location, sonographic characteristics, and vascularity. Lymph node size was recorded using three orthogonal diameters in millimeters (mm). For volume calculation, the linear measurements were converted to centimeters, and lymph node volume was calculated as V = πabc/6, where V denotes volume in cm³, and a, b, and c represent the three orthogonal diameters after conversion to centimeters. Additional imaging examinations, including CT, MRI, or other modalities as clinically indicated, were performed when ultrasound findings were inconclusive for lesion characterization, when deep cervical structural invasion was suspected, or when distant metastasis could not be excluded based on ultrasound and clinical assessment. Laboratory tests included thyroglobulin (Tg), anti-thyroglobulin antibody (TgAb), thyroid function, complete blood count, and coagulation profile.

**Figure 1:**
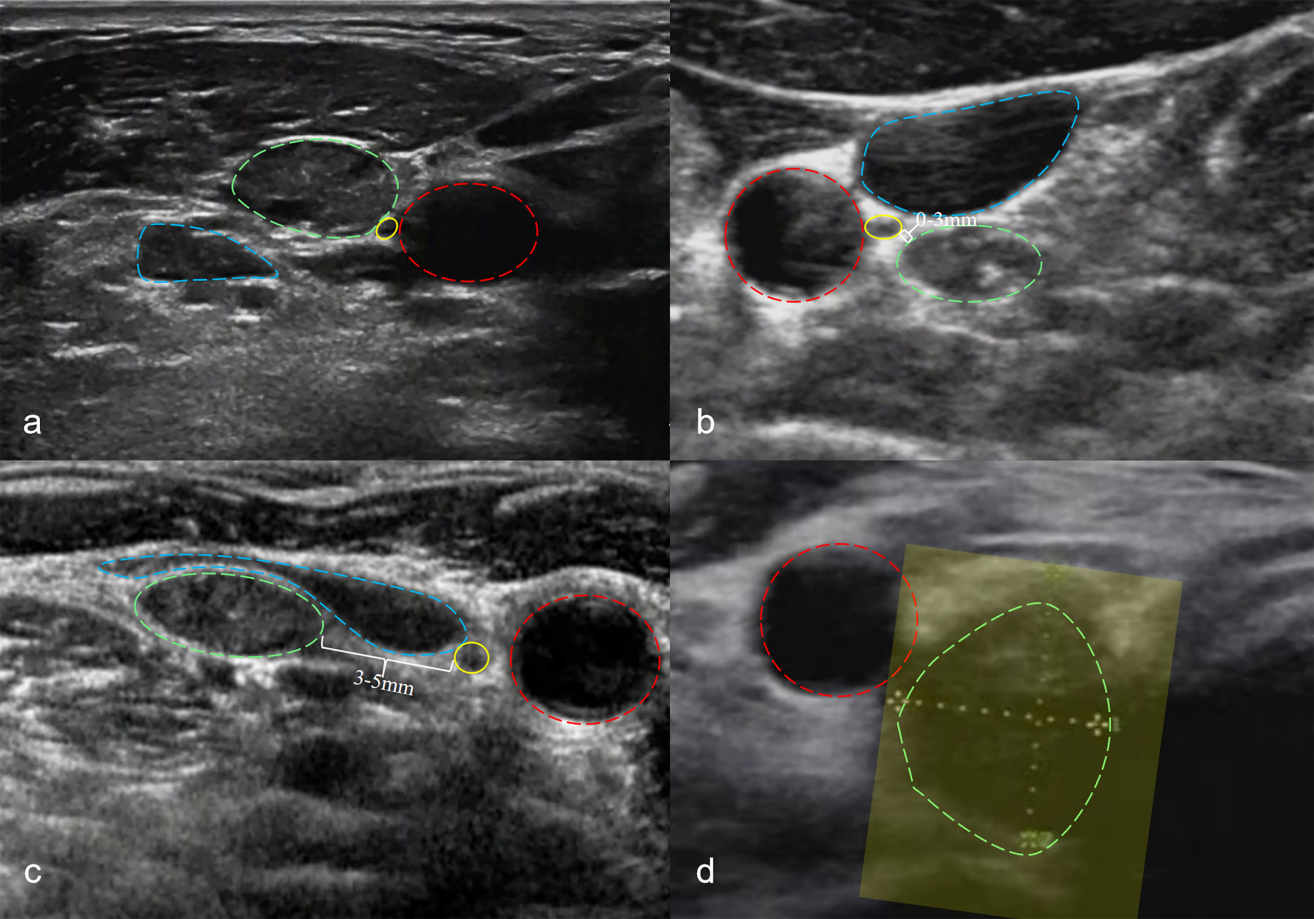
Schematic illustration of four anatomical relationships between cervical metastatic lymph nodes and nerves. The yellow line indicates nerve; the red line indicates artery; the blue line indicates vein; the green outline delineates the contour of the lymph node; and the white line represents the measured minimum distance. The yellow shaded area denotes the projected anatomical pathway of the nerve. **(a)** Type I: the nerve is in direct contact with the lymph node, with a minimum linear distance of 0 mm. **(b)** Type II: the nerve is visualized, and the minimum linear distance between the lymph node and the nerve is >0 and <3 mm, without direct nerve contact or encasement. **(c)** Type III: the nerve is visualized, and the minimum linear distance between the lymph node and the nerve is 3–5 mm. **(d)** Type IV: the nerve is poorly visualized on ultrasound, but the recurrent lymph node is located along the projected anatomical pathway of the nerve based on established cervical landmarks.

Medical records and ultrasound (US) images of all candidate patients were independently reviewed by two ultrasonographers with more than 10 years of experience in thyroid imaging. Disagreements were resolved by a third senior expert. None of these physicians participated in patient treatment. Based on a comprehensive reassessment of archived images and original clinical reports, cases with ultrasound features suggestive of gross extranodal extension (ENE) were excluded, and both sides of the neck were systematically reassessed to rule out additional nodal disease. For the final included ultrasound images, the shortest distance between the capsule of the recurrent lymph node and the outer margin of the adjacent cervical nerve was measured in three orthogonal planes, and the minimum value was recorded. Neural proximity relationships were classified as follows (**Figure 1**): (1) Type I: no intervening fat plane was visible, and the lymph node was closely attached to or circumferentially encased the nerve; (2) Type II: the nerve was visualized, and the shortest distance between the lymph node and the nerve was >0 and <3 mm, without direct attachment or encasement; (3) Type III: the nerve was visualized, and the shortest distance between the LN and the nerve was 3–5 mm; and (4) Type IV: the nerve was not clearly visualized on ultrasound, but the lymph node could be reliably localized along the projected anatomical pathway of the relevant nerve based on predefined cervical anatomical landmarks, including the tracheoesophageal groove for the recurrent laryngeal nerve, the carotid sheath for the vagus nerve, the posterior cervical triangle for the accessory nerve, and the posteromedial carotid sheath/prevertebral region for the cervical sympathetic trunk.

### 2.3 Radiofrequency ablation procedure

RFA was performed by two ultrasonographers with more than 5 years of experience in interventional oncology according to a standardized institutional protocol, mainly including hydrodissection, real-time ultrasound-guided ablation, immediate CEUS confirmation, and perioperative monitoring.

To fully expose the working field, patients were placed in the supine position with neck extension. RFA procedure time was defined as the interval from electrode insertion into the target lesion to completion of ablation, excluding preprocedural preparation and anesthesia. Before ablation, the three diameters of the target lymph node were measured precisely under ultrasound, and color Doppler was used to evaluate vessels along the puncture route to reduce bleeding risk. After local anesthesia with 1% lidocaine, a 21G fine needle connected to an extension tube and syringe was inserted. Under ultrasound guidance, the needle was advanced along a safe anatomical plane while avoiding major vessels and nerve bundles. According to the lymph node location, size, and proximity to critical structures, 5% dextrose solution was preferentially injected into the perineural or perivascular space. Continuous hydrodissection was performed along the lymph node capsule to create a circumferential protective fluid barrier as much as possible, allowing adequate separation of surrounding tissues, especially linear hyperechoic nerve bundles, from the target lymph node. Continuous infusion was used to maintain the fluid barrier and to preserve a safe fluid distance of ≥5 mm between the target lymph node and adjacent critical structures whenever feasible. The total injection volume was approximately 30–60 mL, depending on the local anatomical conditions.

A monopolar internally cooled radiofrequency electrode (Curaway^®^ CRS2000, 18G, 5 mm/7 mm active tip) was then used for ablation. The initial power was set at 10–15 W and adjusted according to lesion size, location, impedance changes, and real-time ultrasound findings of ablation-zone expansion. Prevention of thermal spread was prioritized throughout the procedure. For highly vascular lymph nodes, power could be cautiously increased under close monitoring, while nerve protection remained the primary principle. Larger or irregularly shaped lesions, usually with a diameter >5 mm, were treated using the moving-shot multipoint technique to ensure complete coverage. In contrast, smaller well-defined lesions ≤5 mm, or lesions located in anatomically constrained areas, particularly those adjacent to critical neural structures, were treated using fixed ablation to allow more precise energy delivery. When a vaporization hyperechoic zone appeared around the active tip of the electrode, that area was considered adequately ablated, and the electrode was then repositioned to an adjacent untreated area for stepwise sectional ablation. Ablation was initiated from the deep portion and the nerve-adjacent side of the lesion and then gradually advanced toward the superficial portion. When the safety margin permitted, the ablation zone was extended approximately 2 mm beyond the lesion margin; on the nerve-adjacent side, avoidance of thermal injury was prioritized.

**Figure 2:**
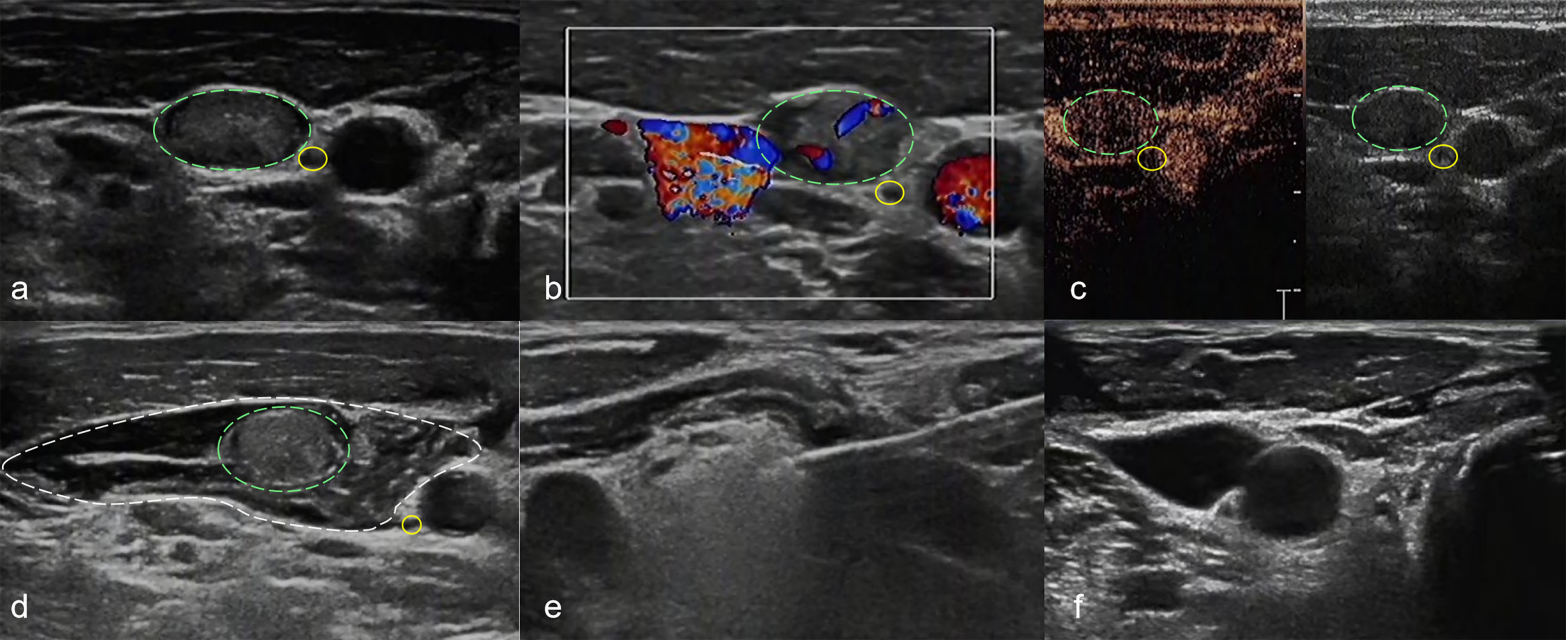
Representative case of RFA for recurrent cervical LNM in the PNR after surgery in a male patient with PTC. **(a)** Pre-ablation ultrasound shows the target metastatic lymph node in the cervical PNR (green outline) adjacent to the nerve (yellow outline). **(b)** CDFI shows focal intralesional blood flow signals within the target lesion. **(c)** Pre-ablation CEUS shows reduced enhancement of the target lesion and no enhancement of the adjacent nerve. **(d)** Hydrodissection creates a protective fluid separation (white dashed line), especially around the adjacent nerve. **(e)** During ablation, the electrode tip is carefully positioned within the lymph node. **(f)** At 6-month follow-up after RFA, the target lesion has completely disappeared.

Immediate CEUS was performed 2–3 min after ablation, and the ablation zone was continuously observed for another 2–3 min to cover the contrast inflow and washout phases. Complete ablation was defined as the absence of enhancement within the ablation zone during both the arterial and venous phases. Residual enhancement was assessed qualitatively rather than by a fixed quantitative threshold. Any focal, nodular, irregular, or peripheral enhancement within or at the margin of the ablation zone was considered residual enhancement, suggesting possible viable tumor tissue. If residual enhancement was detected, supplementary ablation was performed immediately until CEUS showed complete absence of enhancement in the target area (**Figure 2**). Standard bedside monitoring was used to continuously monitor vital signs during the procedure, and color Doppler ultrasound was used to minimize vascular injury. If voice change, severe pain, vasovagal reaction (acute decrease in heart rate or blood pressure), Horner’s syndrome, respiratory or cardiac abnormality, or an expanding hematoma causing tracheal compression occurred during ablation, the procedure was immediately discontinued and appropriate management was initiated. After ablation, manual compression was applied for 20 min to reduce bleeding risk, followed by 12 h of observation. If hematoma was suspected, immediate ultrasound evaluation and compression were performed; if airway compromise was suspected, airway-protective measures, including oxygen supplementation and emergency airway management when necessary, were promptly initiated.

### 2.4 Reoperation procedure

RO was performed under general anesthesia by senior thyroid surgeons with more than 15 years of experience in thyroid surgery. Before surgery, the location and extent of LNM were determined comprehensively based on cervical ultrasound, contrast-enhanced CT or MRI, FNA/CNB results, prior surgical records, and anatomical lesion distribution. The surgical strategy was based on the principles of the 2025 American Thyroid Association (ATA) guidelines for the management of differentiated thyroid cancer (2), emphasizing compartment-oriented cervical lymph node dissection, preoperative imaging–based surgical planning, and preservation of critical neurovascular structures.

Patients were placed in the supine position with a pillow under the shoulders and mild neck extension. After general anesthesia, routine sterilization and draping were performed. The surgical field was entered through the previous surgical scar or a low transverse cervical incision. The skin, subcutaneous tissue, and platysma were incised layer by layer, and skin flaps were raised to expose the relevant anterior and lateral cervical anatomical structures. Dissection was then performed along anatomical planes to enter the planned dissection field, and the extent of dissection was determined according to preoperative imaging, prior surgical records, and intraoperative exploration. For lesions confined to a single compartment, selective lymph node dissection of the corresponding compartment was performed. For lesions involving multiple contiguous compartments or showing more extensive involvement intraoperatively, a broader modified neck dissection was performed. The dissection field included the central compartment (level VI) and/or lateral neck compartments (levels II–V).

Complete dissection was defined as removal of all clinically, radiologically, or intraoperatively suspicious metastatic lymph nodes within the planned dissection compartments, with no gross residual disease identified at the end of surgery. During the procedure, critical structures, including the RLN, VN, and AN, were routinely identified and preserved, with careful protection of the parathyroid glands and their vascular supply. Intraoperative nerve monitoring was used when available. After completion of lymph node dissection, meticulous hemostasis was achieved, the surgical field was confirmed to be free of active bleeding, and the incision was closed layer by layer. Negative-pressure drainage was placed when appropriate according to the surgical field. Postoperatively, vital signs, drainage volume, serum calcium, and parathyroid function were routinely monitored to evaluate early complications and perioperative safety.

### 2.5 Post-treatment assessment and follow-up

Postoperative follow-up was performed systematically at 1, 3, and 6 months after treatment, followed by risk-adapted follow-up every 6–12 months until 24 months of follow-up was completed. Shorter intervals of approximately 6 months were used for patients with higher-risk features or indeterminate findings, whereas longer intervals of up to 12 months were used for patients with stable imaging and biochemical responses. Follow-up assessments included clinical symptom evaluation and imaging examinations. US and CEUS were primarily used to monitor the size, volume changes, and vascularity of the ablation zone and to detect local residual disease and recurrent LNM.

The volume reduction rate (VRR) was calculated as: VRR = (initial volume − final follow-up volume)/initial volume. Complete disappearance after RFA was defined as the absence of any visible target lesion or residual structural abnormality at the original treated site on follow-up imaging, and the complete disappearance rate (CDR) was defined as the proportion of lesions achieving complete disappearance. When the ablation zone increased in size compared with the previous examination, or when a new or enlarged solid component, suspicious enhancement or vascularity, Tg elevation unexplained by locoregional disease, or suspicious cervical LNM was detected, CNB could be performed on the center, margin, and adjacent tissue of the ablation area for confirmation. If distant metastasis was suspected because of unexplained Tg elevation or suggestive clinical symptoms, such as bone pain or respiratory symptoms, further evaluation with CT, PET-CT, or bone scintigraphy was performed to assess disease progression.

### 2.6 Study endpoints

The primary endpoints were local tumor progression (LTP) control and the incidence of treatment-related complications. LTP was defined as any of the following: (1) a newly detected or enlarged suspicious malignant cervical lymph node during follow-up, showing typical malignant imaging features, such as loss of the fatty hilum, microcalcification, cystic change, or peripheral vascularity, and confirmed by FNA or CNB when feasible; or (2) biopsy-proven residual or recurrent viable tumor within or at the margin of the ablation zone. Treatment-related complications were classified as nerve-related and non-nerve-related complications. Nerve-related complications included choking/coughing, vocal cord paralysis, Horner’s syndrome, and shoulder dysfunction. Non-nerve-related complications included transient or permanent hypoparathyroidism (HP), pulmonary infection, and chyle leakage. All nerve-related functional disorders were classified as transient or permanent according to recovery within 6 months: disorders that recovered within 6 months were defined as transient, whereas those persisting beyond 6 months after treatment were defined as permanent.

Hoarseness was defined as new-onset unilateral or bilateral vocal fold mobility impairment after treatment, confirmed by laryngoscopy, including clearly reduced abduction or adduction compared with the preoperative status or the contralateral side, or complete vocal fold immobility. Choking/coughing was defined as persistent cough, choking, or aspiration symptoms newly occurring after treatment and related to nerve irritation or injury. Horner’s syndrome was defined as ipsilateral ptosis, miosis, and/or facial anhidrosis caused by cervical sympathetic chain involvement. Shoulder dysfunction was defined as shoulder abduction weakness, shoulder girdle pain, or limited shoulder range of motion consistent with accessory nerve involvement. HP was defined as a decrease in serum calcium level below the lower limit of the institutional reference range, accompanied by inappropriately low or normal serum parathyroid hormone (PTH) levels relative to the degree of hypocalcemia, with or without the need for calcium and/or vitamin D supplementation. Postoperative parathyroid function was evaluated using serum calcium levels, serum PTH levels, and the requirement for calcium and/or vitamin D supplementation. Recovery within 6 months was considered transient dysfunction, whereas persistent need for long-term calcium supplementation beyond 6 months was defined as permanent HP. Secondary endpoints included treatment-related parameters reflecting procedural efficiency and invasiveness, such as hospital stay and procedure time, because these parameters reflect perioperative recovery burden, procedural complexity, and medical resource utilization. Other secondary endpoints included the volume reduction rate (VRR) of ablated lymph nodes and changes in postoperative parathyroid function.

### 2.7 Statistical analyses

Statistical analyses were performed using R software (version 4.5.1). Continuous variables were expressed as mean ± standard deviation or median (interquartile range), according to their distribution, and categorical variables were presented as frequencies and percentages. For between-group comparisons, normally distributed continuous variables were analyzed using the t test, whereas skewed continuous variables were analyzed using the Wilcoxon rank-sum test. Categorical variables were compared using the χ² test when expected frequencies were sufficient and Fisher’s exact test when expected frequencies were small. Time-to-event outcomes were analyzed using the Kaplan–Meier method and compared using the log-rank test.

Because treatment allocation was nonrandomized, propensity score matching (PSM) was performed to reduce selection bias and improve the balance of observed baseline characteristics between the RFA and RO groups. Propensity scores were estimated using a logistic regression model including age, sex, maximum lesion diameter, lesion location, adjacent nerve type, anatomical subtype, Tg level, and TgAb status. Patients were matched at a 1:1 ratio using the nearest-neighbor method without replacement. Post-matching balance was assessed using standardized mean differences and between-group comparisons.

The interaction between treatment modality and anatomical subtype was explored using regression models including treatment modality, anatomical subtype, and their interaction term, and was evaluated using the likelihood ratio test. To control inflation of type I error due to multiple comparisons, P values for subgroup analyses were adjusted using the Holm method; overall between-group comparisons and primary outcome analyses were not adjusted for multiple comparisons. All tests were two-sided, and P<0.05 was considered statistically significant. Missing data was uncommon and was handled using complete-case analysis; no data imputation was performed.

## 3 Results

### 3.1 Demographic and lymph-node-related characteristics

**Figure 3:**
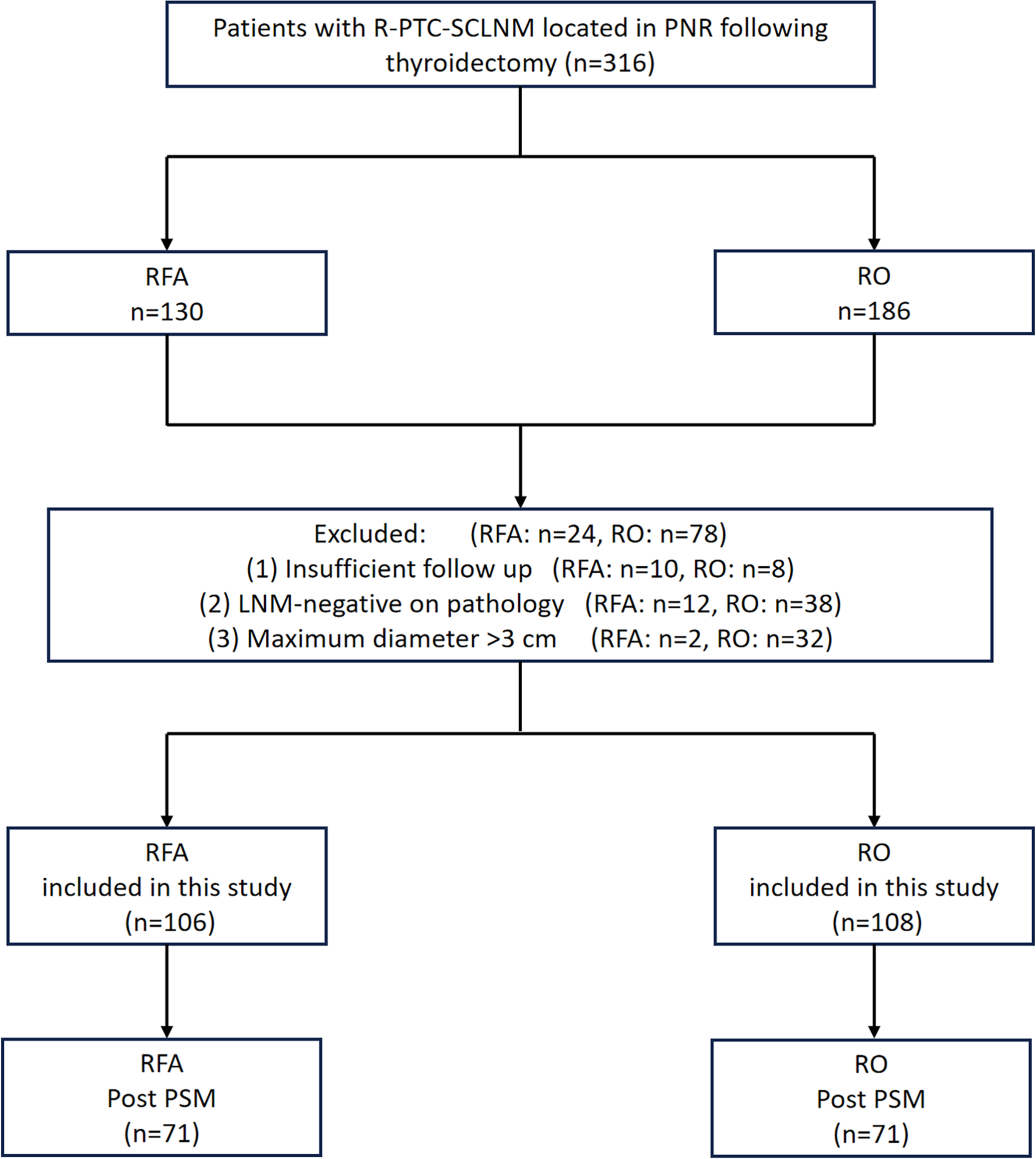
Flowchart of the inclusion and exclusion criteria applied in this study.

**Table 1** summarizes the demographic and tumor-related characteristics of the two groups before and after PSM. Between January 2018 and December 2022, a total of 316 patients with R-PTC-SCLNM located in PNRs underwent RFA or RO. Among them, 102 patients were excluded because they did not meet the inclusion criteria. Finally, 214 patients were included in the initial analysis, including 106 in the RFA group and 108 in the RO group. In the RFA group, 82.1% (87/106) of patients refused RO, and 17.9% (19/106) had RO-related contraindications. Before PSM, the two groups differed significantly in age, maximum lesion diameter, and involved cervical lymph node compartment. After PSM, 71 matched pairs were obtained. Therefore, 142 patients were included in the final analysis (**Figure 3**).

**Table 1:** Demographic and tumor-related characteristics before and after PSM.

Parameter		Before PSM		P value	After PSM		P value
		RFA (n = 106)	RO (n = 108)		RFA (n = 71)	RO (n = 71)	
Gender	Male	36 (34.0)	46 (42.6)	0.194	25 (35.2)	28 (39.4)	0.729
	Female	70 (66.0)	62 (57.4)		46 (64.8)	43 (60.6)	
Age (years)		44.59 ± 15.28	49.56 ± 14.19	0.007**	47.01 ± 15.33	47.28 ± 14.60	0.915
Maximum lesion diameter (mm)		9.49 ± 4.34	11.69 ± 6.45	0.011*	10.1 ± 4.65	10.69 ± 5.98	0.513
Tg (ng/mL)		7.82 ± 16.34	7.75 ± 11.21	0.971	8.37 ± 14.78	7.19 ± 10.01	0.579
TgAb status	Positive	10 (9.4)	10 (9.3)	1.000	5 (7.0)	8 (11.3)	0.561
	Negative	96 (90.6)	98 (90.7)		66 (93.0)	63 (88.7)	
Location	II	11 (10.4)	6 (5.6)	0.040*	3 (4.2)	3 (4.2)	0.963
	III	29 (27.4)	18 (16.7)		18 (25.4)	15 (21.1)	
	IV	23 (21.7)	38 (35.2)		20 (28.2)	22 (31.0)	
	V	1 (0.9)	2 (1.8)		1 (1.4)	2 (2.8)	
	VI	37 (34.9)	43 (39.8)		29 (40.8)	29 (40.8)	
	VII	5 (4.7)	1 (0.9)		0 (0)	1 (1.4)	
Adjacent Nerve Type	RLN	40 (37.7)	43 (39.8)	0.492	29 (40.8)	29 (40.8)	1.000
	VN	58 (54.7)	51 (47.2)		35 (49.3)	34 (47.9)	
	AN	2 (1.9)	5 (4.6)		2 (2.8)	2 (2.8)	
	CST	6 (5.7)	9 (8.3)		5 (7.0)	6 (8.5)	
Nerve–lesion relationship	Type I	11 (10.4)	20 (18.5)	0.280	6 (8.5)	7 (9.9)	0.866
	Type II	43 (40.6)	36 (33.3)		25 (35.2)	29 (40.8)	
	Type III	29 (27.4)	25 (23.1)		19 (26.8)	16 (22.5)	
	Type IV	23 (21.7)	27 (25.0)		21 (29.6)	19 (26.8)	

Data are presented as mean ± SD or n (%).

*P<0.05; **P<0.01.

### 3.2 Intraoperative and postoperative outcomes

Treatment-related intraoperative and postoperative variables are shown in **Table 2**. In the RFA group, all 71 patients with nerve-adjacent R-PTC-SCLNM completed ablation in a single session, and immediate post-ablation CEUS showed complete absence of enhancement in all target lesions, yielding a technical success rate of 100%. In the RO group, all 71 patients successfully underwent repeat cervical lymph node dissection, including lateral neck dissection in 18 patients (25.4%), central compartment dissection in 48 patients (67.6%), and combined central and lateral neck dissection in 5 patients (7.0%), with a surgical success rate of 100%. Compared with RO, RFA was associated with shorter procedure time (62.13 ± 36.35 min vs. 144.59 ± 64.78 min, P<0.001), shorter hospital stay (1.07 ± 0.26 days vs. 10.24 ± 4.42 days, P<0.001), less intraoperative blood loss (1.60 ± 0.18 g vs. 48.03 ± 31.33 g, P<0.001), and required only a 1–2 mm skin puncture incision. Subgroup analysis showed that, except for procedure time in Type I lesions, which did not reach statistical significance, these minimally invasive advantages were generally consistent across anatomical subtypes.

**Table 2:** Comparison of treatment variables after RFA and RO.

Anatomical subtype	Group	n	Procedure time (min)	Hospitalization time (d)	Estimated blood loss (g)	Incision length (mm)
Total	RFA	71	62.13 ± 36.35	1.07 ± 0.26	1.60 ± 0.18	1.11 ± 0.23
	RO	71	144.59 ± 64.78	10.24 ± 4.42	48.03 ± 31.33	96.21 ± 41.55
	P value		< 0.001	< 0.001	< 0.001	< 0.001
Type I	RFA	6	86.67 ± 70.52	1.17 ± 0.41	1.62 ± 0.18	1.07 ± 0.28
	RO	7	120.29 ± 50.24	6.43 ± 2.44	38.57 ± 12.15	61.39 ± 34.81
	P value		0.338	0.003	< 0.001	< 0.001
Type II	RFA	25	56.56 ± 26.78	1.08 ± 0.28	1.60 ± 0.18	1.14 ± 0.25
	RO	29	161.55 ± 77.95	10.83 ± 3.56	52.07 ± 38.65	106.61 ± 42.73
	P value		< 0.001	< 0.001	< 0.001	< 0.001
Type III	RFA	19	73.89 ± 38.26	1.05 ± 0.23	1.62 ± 0.17	1.10 ± 0.29
	RO	16	133.94 ± 46.51	10.25 ± 3.99	49.38 ± 21.75	109.88 ± 33.32
	P value		< 0.001	< 0.001	< 0.001	< 0.001
Type IV	RFA	21	51.10 ± 26.93	1.05 ± 0.22	1.56 ± 0.19	1.12 ± 0.15
	RO	19	136.63 ± 58.00	10.74 ± 5.88	44.21 ± 31.19	81.56 ± 38.94
	P value		< 0.001	< 0.001	< 0.001	< 0.001

Data are presented as mean ± SD. P values were calculated using the Student’s t test or Mann–Whitney U test, as appropriate.

### 3.3 Volume changes of metastatic lymph node lesions before and after ablation

**Figure 4:**
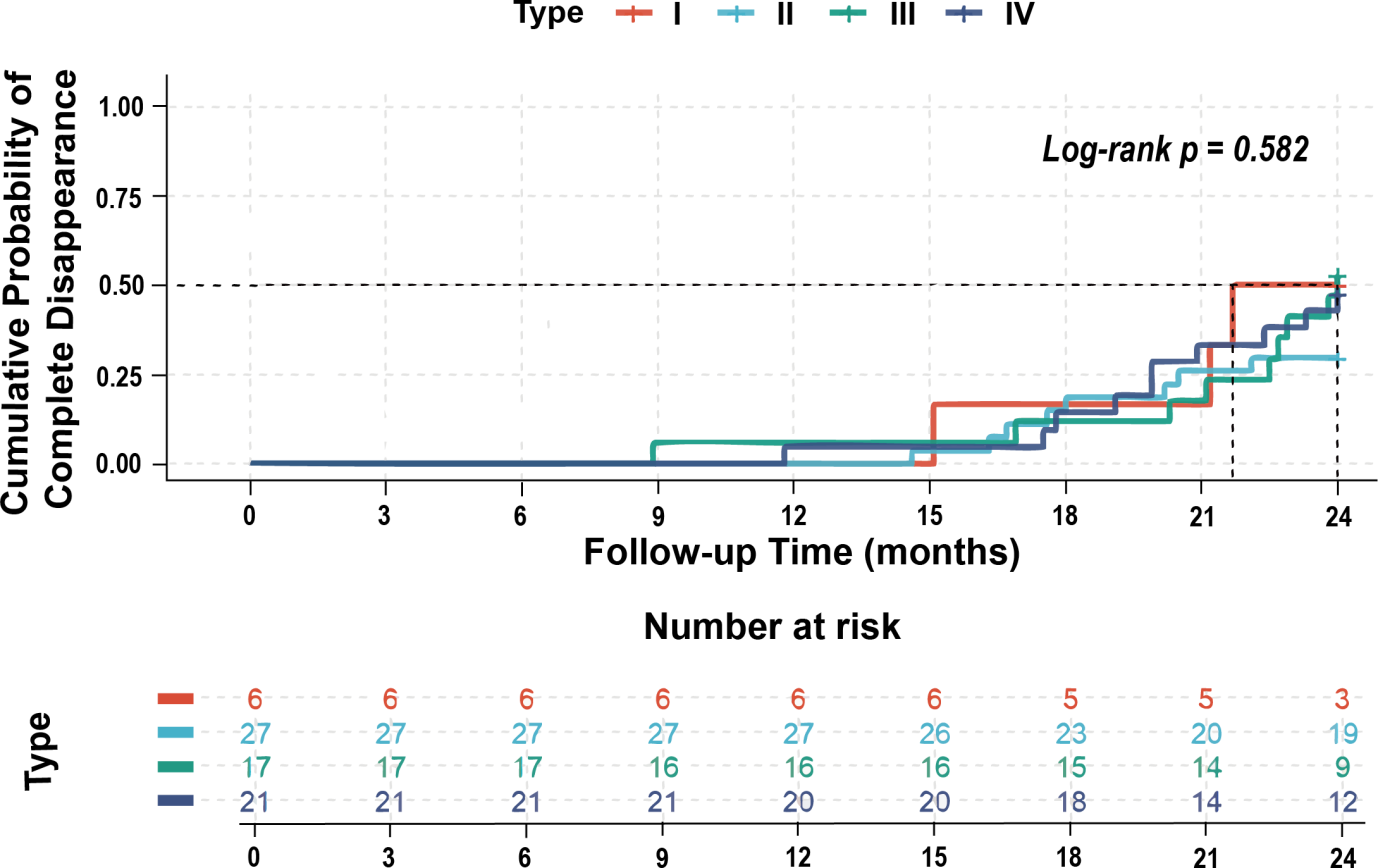
Kaplan–Meier analysis of the cumulative probability of complete disappearance of metastatic lymph nodes after RFA by anatomical subtype. The cumulative probability of complete disappearance was estimated as 1 minus the Kaplan–Meier persistence function. Differences among anatomical subtypes were compared using the log-rank test. No significant difference was observed among the four subtypes (log-rank χ²=1.96, P = 0.582).

Dynamic changes in the target lesions after RFA are shown in **Table 3**. During follow-up, both the maximum diameter and volume of the target lesions showed a continuous decreasing trend. At the 24-month follow-up, the mean maximum diameter decreased from 10.10 ± 4.65 mm at baseline to 1.98 ± 2.11 mm, and the mean volume decreased from 0.27 ± 0.44 cm³ to 0.01 ± 0.02 cm³, both of which were significantly lower than baseline (both P<0.001). The mean volume reduction ratio (VRR) reached 0.97 ± 0.04. At the final follow-up, 42.3% (30/71) of metastatic lymph nodes had completely disappeared, with a median disappearance time of 20.3 months. Subgroup analysis showed that lesions in all four anatomical subtypes continued to shrink during follow-up, and the VRR gradually increased over time (**Table 3**). Kaplan–Meier analysis showed no significant difference in the cumulative complete disappearance rate of LNM among anatomical subtypes (log-rank χ²=1.96, P = 0.582; **Figure 4**). Although not statistically significant, Type II lesions showed a numerically lower cumulative probability of complete disappearance than the other subtypes.

**Table 3:** Changes in lesion diameter, volume, and VRR after RFA across follow-up visits.

Follow-up	Parameter	Total	Type I	Type II	Type III	Type IV
Before RFA	Mean Largest Diameter (mm)	10.10 ± 4.65	8.10 ± 2.61	9.87 ± 4.32	9.56 ± 3.7	11.4 ± 5.96
	Volume (cm³)	0.27 ± 0.44	0.16 ± 0.17	0.26 ± 0.26	0.16 ± 0.15	0.42 ± 0.76
After RFA						
3 months	Mean Largest Diameter (mm)	8.66 ± 4.05*	7.35 ± 2.07*	8.26 ± 3.75*	8.29 ± 3.53*	9.85 ± 5.07*
	Volume (cm³)	0.18 ± 0.31*	0.12 ± 0.13*	0.18 ± 0.18*	0.11 ± 0.11*	0.28 ± 0.54*
	VRR	0.34 ± 0.09*	0.30 ± 0.06*	0.34 ± 0.10*	0.33 ± 0.06*	0.34 ± 0.09*
6 months	Mean Largest Diameter (mm)	7.29 ± 3.33*	6.12 ± 2.01*	6.90 ± 3.05*	7.07 ± 2.86*	8.3 ± 4.17*
	Volume (cm³)	0.12 ± 0.19*	0.08 ± 0.10*	0.11 ± 0.11*	0.07 ± 0.07*	0.18 ± 0.32*
	VRR	0.56 ± 0.10*	0.56 ± 0.06*	0.58 ± 0.09*	0.55 ± 0.13*	0.56 ± 0.09*
9 months	Mean Largest Diameter (mm)	5.96 ± 3.05*	4.70 ± 1.92*	5.54 ± 2.55*	5.86 ± 3.31*	6.94 ± 3.56*
	Volume (cm³)	0.07 ± 0.12*	0.04 ± 0.05*	0.06 ± 0.06*	0.04 ± 0.04*	0.11 ± 0.20*
	VRR	0.77 ± 0.09*	0.77 ± 0.07*	0.77 ± 0.08*	0.79 ± 0.09*	0.75 ± 0.09*
12 months	Mean Largest Diameter (mm)	4.50 ± 2.38*	3.78 ± 1.68*	4.22 ± 1.85*	4.15 ± 2.37*	5.36 ± 3.00*
	Volume (cm³)	0.04 ± 0.06*	0.03 ± 0.03*	0.03 ± 0.03*	0.02 ± 0.03*	0.06 ± 0.10*
	VRR	0.87 ± 0.08*	0.87 ± 0.06*	0.89 ± 0.06*	0.88 ± 0.10*	0.85 ± 0.10*
18 months	Mean Largest Diameter (mm)	3.27 ± 2.27*	2.62 ± 1.72*	3.06 ± 2.06*	3.15 ± 2.18*	3.81 ± 2.72*
	Volume (cm³)	0.02 ± 0.04*	0.01 ± 0.02*	0.02 ± 0.02*	0.01 ± 0.02*	0.04 ± 0.06*
	VRR	0.94 ± 0.05*	0.94 ± 0.04*	0.94 ± 0.04*	0.94 ± 0.07*	0.93 ± 0.05*
24 months	Mean Largest Diameter (mm)	1.98 ± 2.11*	1.13 ± 1.40*	2.18 ± 1.75*	1.57 ± 2.04*	2.30 ± 2.70*
	Volume (cm³)	0.01 ± 0.02*	0.003 ± 0.007*	0.01 ± 0.01*	0.006 ± 0.009*	0.02 ± 0.04*
	VRR	0.97 ± 0.04*	0.99 ± 0.01*	0.97 ± 0.04*	0.97 ± 0.05*	0.98 ± 0.03*

Data are presented as mean ± SD.

*P<0.05 compared with baseline.

### 3.4 Treatment outcomes

**Figure 5:**
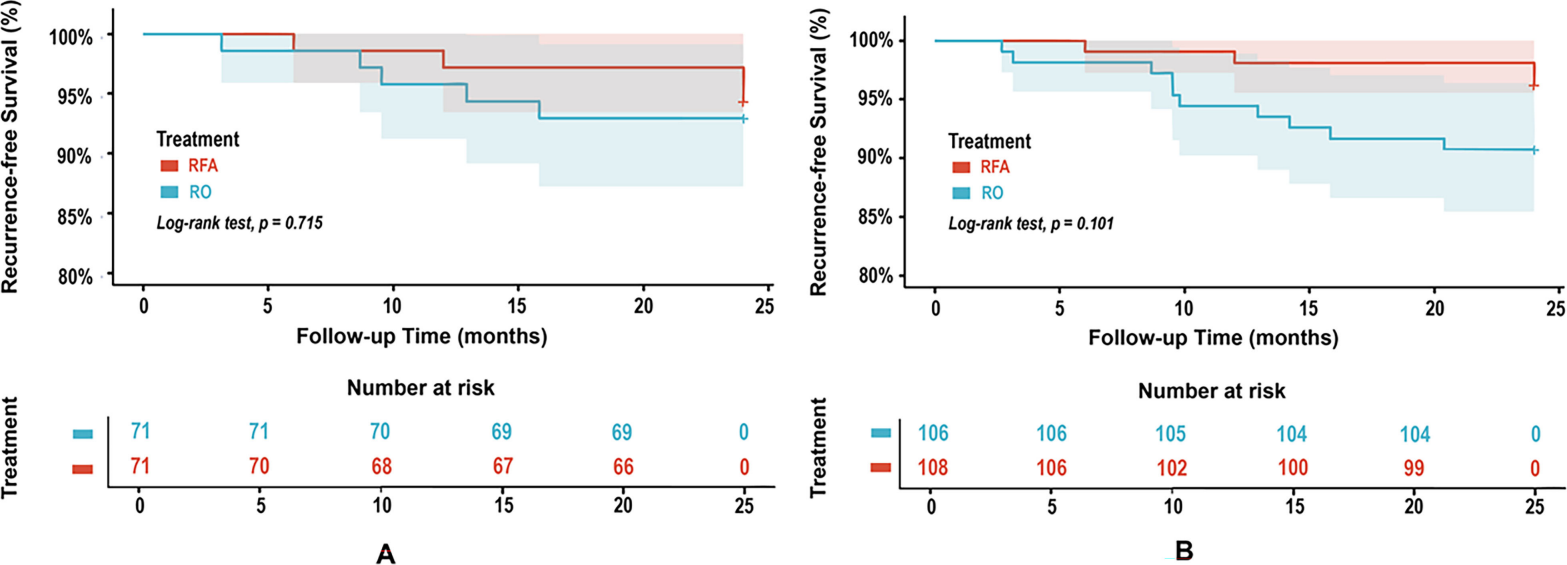
Kaplan–Meier curves comparing recurrence-free survival between the RFA and RO groups before and after PSM. **(A)** Before PSM. **(B)** After PSM. Curves were compared using the log-rank test. No significant difference was observed between the RFA and RO groups before PSM (log-rank χ²=2.694, P = 0.101) or after PSM (log-rank χ²=0.134, P = 0.715). The red and blue shaded areas indicate the 95% confidence intervals for the RFA and RO groups, respectively. The lower panels show the number at risk.

Local recurrence rates before and after PSM are summarized in **Table 4**. After PSM, the overall local recurrence rate was lower in the RFA group than in the RO group, but the difference was not statistically significant (5.6% vs. 7.0%, P = 1.000). Within each anatomical subtype, no statistically significant difference in recurrence rate was observed between the RFA and RO groups. Given the limited sample size in some subtypes after PSM, particularly Type I lesions, pre-PSM subgroup results were included as a sensitivity analysis, showing trends generally consistent with those in the matched cohort. Further analysis showed no significant interaction between treatment modality and anatomical subtype for local recurrence before PSM (P for interaction=0.822) or after PSM (P for interaction=0.914). Kaplan–Meier analysis (**Figure 5**) showed no statistically significant difference in local recurrence-free survival between the two groups after PSM (log-rank χ²=0.134, P = 0.715). The pre-PSM cohort also showed no statistically significant difference between groups (log-rank χ²=2.694, P = 0.101), consistent with the recurrence-rate analysis.

**Table 4:** Comparison of local recurrence rates between the RFA and RO groups before and after PSM.

Anatomical subtype	Recurrence rate, n/N (%)		P value
	RFA	RO	
Before PSM			
Total	4/106 (3.8)	10/108 (9.3)	0.165
Type I	1/11 (9.1)	2/20 (10.0)	1.000
Type II	1/43 (2.3)	4/36 (11.1)	0.687
Type III	1/29 (3.4)	2/25 (8.0)	1.000
Type IV	1/23 (4.3)	2/27 (7.4)	1.000
After PSM			
Total	4/71 (5.6)	5/71 (7.0)	1.000
Type I	1/6 (16.7)	0/7 (0)	1.000
Type II	1/25 (4.0)	4/29 (13.8)	1.000
Type III	1/19 (5.3)	1/16 (6.3)	1.000
Type IV	1/21 (4.8)	0/19 (0)	1.000

Data are shown as n/N (%). P values for subgroup analyses were adjusted using the Holm method.

After PSM, nine patients developed second recurrence, including four in the initial RFA group, with a median time to second recurrence of 18.0 months, and five in the initial RO group, with a median time to second recurrence of 9.53 months. Third-line management was individualized according to lesion characteristics and clinical assessment. In the initial RFA group, the four patients underwent second thermal ablation, repeat cervical lymph node dissection for multifocal disease with complex anatomy, ^125^I seed brachytherapy, or active surveillance, respectively. In the initial RO group, two patients underwent a third cervical lymph node dissection, and three underwent active surveillance. After these third-line interventions or surveillance, no new local recurrence or distant metastasis was observed during follow-up (**Supplementary Table 1**).

### 3.5 Complications

Complication rates after PSM are summarized in **Table 5**. The overall complication rate was significantly lower in the RFA group than in the RO group (11.3% vs. 45.1%, P<0.001), including both nerve-related complications (9.9% vs. 28.2%, P = 0.009) and non-nerve-related complications (1.4% vs. 16.9%, P = 0.002).

**Table 5:** Occurrence of complications in the RFA and RO groups after PSM.

Complication type	Total (n=142)		P value	Type I (n=13)		P value	Type II (n=54)		P value	Type III (n=35)		P value	Type IV (n=40)		P value
	RFA n=71	RO n=71		RFA n=6	RO n=7		RFA n=25	RO n=29		RFA n=19	RO n=16		RFA n=21	RO n=19	
Overall complications	8 (11.3)	32 (45.1)	<0.001	0 (0)	4 (57.1)	0.210	3 (12.0)	18 (62.1)	<0.001	1 (5.3)	4 (25.0)	0.312	4 (19.0)	6 (31.6)	0.473
Nerve-related complications	7 (9.9)	20 (28.2)	0.009	0 (0)	3 (42.9)	0.577	3 (12.0)	10 (34.5)	0.259	1 (5.3)	3 (18.8)	0.624	3 (14.3)	4 (21.1)	0.689
Transient hoarseness	4 (5.6)	9 (12.7)		0 (0)	2 (28.6)		2 (8.0)	4 (13.8)		0 (0)	1 (6.3)		2 (9.5)	2 (10.5)	
Permanent hoarseness	2 (2.8)	5 (7.0)		0 (0)	1 (14.3)		1 (4.0)	3 (10.3)		0 (0)	0 (0)		1 (4.8)	1 (5.3)	
Transient cough	0 (0)	4 (5.6)		0 (0)	0 (0)		0 (0)	2 (6.9)		0 (0)	1 (6.3)		0 (0)	1 (5.3)	
Permanent cough	0 (0)	0 (0)		0 (0)	0 (0)		0 (0)	0 (0)		0 (0)	0 (0)		0 (0)	0 (0)	
Transient Horner’s syndrome	1 (1.4)	0 (0)		0 (0)	0 (0)		0 (0)	0 (0)		1 (5.3)	0 (0)		0 (0)	0 (0)	
Permanent Horner’s syndrome	0 (0)	1 (1.4)		0 (0)	0 (0)		0 (0)	1 (3.4)		0 (0)	0 (0)		0 (0)	0 (0)	
Transient Shoulder dysfunction	0 (0)	0 (0)		0 (0)	0 (0)		0 (0)	0 (0)		0 (0)	0 (0)		0 (0)	0 (0)	
Permanent Shoulder dysfunction	0 (0)	1 (1.4)		0 (0)	0 (0)		0 (0)	0 (0)		0 (0)	1 (6.3)		0 (0)	0 (0)	
Non-nerve-related complications	1 (1.4)	12 (16.9)	0.002	0 (0)	1 (14.3)	1.000	0 (0)	8 (27.6)	0.021	0 (0)	1 (6.3)	1.000	1 (4.8)	2 (10.5)	1.000
Transient HP	0 (0)	3 (4.2)		0 (0)	0 (0)		0 (0)	2 (6.9)		0 (0)	0 (0)		0 (0)	1 (5.3)	
Permanent HP	0 (0)	3 (4.2)		0 (0)	1 (14.3)		0 (0)	1 (3.4)		0 (0)	1 (6.3)		0 (0)	0 (0)	
Pulmonary infection	1 (1.4)	4 (5.6)		0 (0)	0 (0)		0 (0)	3 (10.3)		0 (0)	0 (0)		1 (4.8)	1 (5.3)	
Chyle leak	0 (0)	2 (2.8)		0 (0)	0 (0)		0 (0)	2 (6.9)		0 (0)	0 (0)		0 (0)	0 (0)	

Data are presented as n (%). P values were calculated using the two-sided Fisher’s exact test. For subgroup analyses by anatomical subtype, P values were adjusted for multiple comparisons using the Holm method.

Subgroup analysis showed that the advantage of RFA was mainly observed in Type II lesions, in which the overall complication rate (12.0% vs. 62.1%, P<0.001) and non-nerve-related complication rate (0% vs. 27.6%, P = 0.021) were significantly lower than those in the RO group. In the remaining subtypes, between-group differences did not reach statistical significance. Although nerve-related complications did not differ significantly between RFA and RO within any anatomical subtype, their incidence was numerically lower in the RFA group across all subtypes.

In the pre-PSM cohort (**Supplementary Table 2**), the RFA group had significantly lower rates of overall complications (9.4% vs. 43.5%, P<0.001), nerve-related complications (8.5% vs. 27.8%, P<0.001), and non-nerve-related complications (0.9% vs. 15.7%, P<0.001) than the RO group, showing a pattern consistent with the matched cohort. In the pre-PSM Type I subgroup, the complication rate was numerically lower in the RFA group, but the difference was not statistically significant. In Type II lesions, however, the RFA group had significantly lower overall, nerve-related, and non-nerve-related complication rates than the RO group. Exploratory interaction analysis showed no significant interaction between treatment modality and anatomical subtype for nerve-related complications either before or after PSM (P for interaction=0.693 and 0.432, respectively).

In addition, to evaluate the safety of third-line treatment after second recurrence, we further analyzed complications associated with subsequent interventions. No severe complications occurred in the RFA group. In the RO group, one patient developed dyspnea and permanent recurrent laryngeal nerve injury after a third surgery (**Supplementary Table 1**).

## 4 Discussion

This study showed that RFA for nerve-adjacent R-PTC-SCLNM achieved a technical success rate of 100%, with complete absence of enhancement in all target lesions on post-ablation CEUS. At the 24-month follow-up, the mean VRR in the RFA group reached 0.97 ± 0.04, and 42.3% of metastatic lymph nodes had completely disappeared, which was generally consistent with the favorable local regression reported in previous studies (12, 14). Regarding treatment outcomes, the overall local recurrence rate did not differ significantly between the RFA and RO groups either before or after PSM, and the Kaplan–Meier analysis showed consistent findings. Subgroup and interaction analyses did not suggest obvious heterogeneity in treatment efficacy. These findings indicate that RFA achieved acceptable local control during the follow-up period of this study. However, because this study was not designed as a non-inferiority or equivalence trial, the long-term oncologic efficacy of RFA requires further validation. Meanwhile, RFA was associated with clear minimally invasive advantages in terms of procedure time, intraoperative blood loss, hospital stay, and incision length, suggesting that it may provide a low-trauma treatment option for patients who are unwilling or unsuitable to undergo reoperation.

Currently, RO remains an important treatment strategy for R-PTC-SCLNM, particularly for patients with multifocal disease, larger lesions, or lesions requiring complete pathological assessment. However, scar adhesion and disruption of normal anatomical planes after previous neck surgery may increase the difficulty of nerve identification and preservation during reoperation. Previous studies have reported transient vocal cord paralysis rates of approximately 5%–10% and permanent vocal cord paralysis rates of approximately 1%–4% after cervical lymph node dissection (15, 16), while the incidence of Horner’s syndrome has been reported to be approximately 0.56% (17). Accessory nerve injury may lead to neck–shoulder syndrome, with recovery requiring 6–12 months in some patients (18). After PSM in the present study, the rate of nerve-related complications was significantly lower in the RFA group than in the RO group (P = 0.009). It should be noted that RFA cannot completely eliminate the risk of nerve injury, particularly when the lesion encases or closely abuts the nerve. Nevertheless, under strict case selection, real-time ultrasound monitoring, and protective ablation strategies, this study did not observe a higher risk of nerve injury with RFA than with RO.

In addition to nerve injury, non-nerve-related complications were also significantly less frequent in the RFA group than in the RO group (P = 0.002), mainly because complications such as hypoparathyroidism, chyle leakage, and postoperative infection occurred more frequently in the RO group, whereas these events were rare or absent in the RFA group. Accordingly, the overall complication rate was significantly lower in the RFA group than in the RO group (P<0.001), consistent with the findings of Lin et al. in recurrent level VI lymph node metastases (19). This safety advantage may be associated with the focal, real-time visualized, and low-trauma nature of RFA, which may reduce tissue traction, dissection, and related injury caused by extensive re-exploration in previously operated regions.

Subgroup analysis suggested that the safety advantage of RFA was most evident in Type II lesions. This may be because these lesions are close to the nerve but often still have an identifiable anatomical interval, allowing safe hydrodissection and dynamic monitoring. In Type III lesions, the distance from the nerve is greater (3–5 mm), providing a relatively wider safety margin, which may reduce the risk of nerve injury in both treatment groups and thereby attenuate between-group differences. In contrast, Type I lesions closely adhere to or encase the nerve, whereas Type IV lesions are located along standard neural pathways but have poorly visualized nerve courses; both situations may increase the technical difficulty of RFA and RO.

Given the limited sample size in some subtypes after PSM, particularly Type I lesions, the pre-PSM cohort was further included as a sensitivity analysis. The pre-PSM results generally supported the direction of the findings in the matched cohort, showing an overall lower complication rate in the RFA group, with the most evident safety difference observed in Type II lesions; however, differences in the other subgroups did not reach statistical significance, suggesting that the current data remain limited in distinguishing outcomes in complex high-risk subtypes, particularly Type I and Type IV lesions. Exploratory interaction analysis showed no significant interaction between treatment modality and anatomical subtype, suggesting that the safety profile of RFA relative to RO did not appear to vary substantially across anatomical subtypes, and that the observed findings were not solely driven by the Type II subgroup. Although some subtype comparisons did not reach statistical significance because of limited sample size and event numbers, no signal of higher nerve injury risk with RFA than with RO was observed in any subtype. Therefore, under strict case selection and standardized protective ablation strategies, RFA may have a potential safety advantage across different nerve-adjacent anatomical subtypes.

The management of second recurrence further suggests that treatment of recurrent LNM requires balancing disease control with cumulative treatment-related risk. After PSM, nine patients developed second recurrence; after individualized third-line treatment or active surveillance, no new local recurrence or distant metastasis was observed. Wu et al. reported that repeat ablation can maintain disease control with an acceptable safety profile (20). In the present study, patients in the RFA group who received active third-line treatment developed no severe complications or further progression, whereas one patient in the RO group developed dyspnea and permanent recurrent laryngeal nerve injury after a third surgery. This single case cannot establish a direct causal relationship; however, it suggests that scar adhesion, fibrosis, and altered anatomical planes after multiple neck surgeries may increase the difficulty of nerve preservation.

This study has several limitations. First, although PSM reduced baseline confounding, it also decreased the effective sample size, particularly in small subgroups such as Type I lesions, which may have limited statistical power. Nevertheless, the sensitivity analysis in the unmatched cohort showed consistent trends, supporting the robustness of the main findings. Second, the retrospective design and stringent inclusion criteria may have selected patients with localized lesions, good ultrasound visibility, and lesions amenable to safe ablation, thereby limiting the generalizability of the findings to multifocal, confluent, larger, or anatomically complex lesions. In addition, parameters such as subcentimeter satellite lesions, histologic aggressiveness, and molecular features were not systematically available, and residual confounding cannot be excluded. Third, the limited follow-up duration restricts the assessment of long-term oncologic safety, delayed recurrence, and long-term neurological functional outcomes. Finally, ENE was mainly assessed by ultrasound, which may not reliably detect microscopic invasion, particularly in the RFA group without histopathological confirmation. Future multicenter, prospective studies with long-term follow-up are needed to further validate these findings.

## 5 Conclusions

Ultrasound-guided RFA demonstrated encouraging safety and short-term efficacy for nerve-adjacent R-PTC-SCLNM. Compared with RO, RFA showed a more favorable complication profile and acceptable short-term local disease control. For patients who are unsuitable for or unwilling to undergo surgery, RFA may serve as a potential minimally invasive treatment option when cases are carefully selected, lesions are clearly visualized, safe hydrodissection is feasible, and the procedure is performed by experienced operators.

## Data Availability

The raw data supporting the conclusions of this article will be made available by the authors, without undue reservation.
