# Supplementary material for: Comparison of ultrasound-guided percutaneous radiofrequency ablation and reoperation for nerve-adjacent cervical lymph node recurrence of papillary thyroid carcinoma: a propensity score–matched study
Source: Front Endocrinol (Lausanne). 2026 Jun 11;17:1852967. doi: 10.3389/fendo.2026.1852967 (PMC13293904; doi:10.3389/fendo.2026.1852967)
Supplement: Supplementary file 1 [file Table1.docx]

**SUPPLEMENTAL TABLE 1. Third-Line Management, Clinical Outcomes, and Major Complications in Patients With Second Recurrence**

|  | **Post-ablation** | | | | |  | **Post-reoperation** | | | | |
| --- | --- | --- | --- | --- | --- | --- | --- | --- | --- | --- | --- |
|  | **Overall** | **STA** | **STS** | **AS** | **BT** |  | **Overall** | **STA** | **TTS** | **AS** | **BT** |
| Number | 4 | 1 | 1 | 1 | 1 |  | 5 | 0 | 2 | 3 | 0 |
| Median Time to Second Recurrence (months) | 18.0 (16.5) |  |  |  |  |  | 9.53  (4.26) |  |  |  |  |
| Major Complications  (n%) | 0 | 0 | 0 | 0 | 0 |  | 1 | 0 | 1 | 0 | 0 |

Data are expressed as median (interquartile range)

STA, second thermal ablation; STS, second surgery; TTS, third surgery; AS, active surveillance; BT, ¹²⁵I seed brachytherapy.

**SUPPLEMENTAL TABLE 2． Occurrence of complications in the RFA and RO groups before PSM**

|  | **Total (n=214)** | | **P value** | **Type I (n=31)** | | **P value** | **Type II (n=79)** | | **P value** | **Type III (n=54)** | | **P value** | **Type IV (n=50)** | | **P value** |
| --- | --- | --- | --- | --- | --- | --- | --- | --- | --- | --- | --- | --- | --- | --- | --- |
|  | **RFA**  **n=106** | **RO**  **n=108** |  | **RFA**  **n=11** | **RO**  **n=20** |  | **RFA**  **n=43** | **RO**  **n=36** |  | **RFA**  **n=29** | **RO**  **n=25** |  | **RFA**  **n=23** | **RO**  **n=27** |  |
| **Overall complications** | 10 (9.4) | 47 (43.5) | <0.001 | 1 (9.0) | 10 (50.0) | 0.094 | 4 (9.3) | 20 (55.6) | <0.001 | 1 (3.4) | 9 (36.0) | 0.01 | 4 (17.3) | 8 (29.6) | 0.345 |
| **Nerve-related complications** | 9 (8.5) | 30 (27.8) | <0.001 | 1 (9.0) | 6 (30.0) | 0.743 | 4 (9.3) | 12 (33.3) | 0.045 | 1 (3.4) | 6 (24.0) | 0.122 | 3 (13.0) | 6 (22.2) | 0.743 |
| Transient hoarseness | 5 (4.7) | 12 (11.1) |  | 0 (0) | 2 (10.0) |  | 2 (4.7) | 6 (16.7) |  | 0 (0) | 1 (4.0) |  | 2 (8.7) | 2 (7.4) |  |
| Permanent hoarseness | 3 (2.8) | 9 (8.3) |  | 0 (0) | 3 (15.0) |  | 2 (4.7) | 3 (6.3) |  | 0 (0) | 1 (4.0) |  | 1 (4.3) | 3 (11.1) |  |
| Transient cough | 0 (0) | 5 (4.6) |  | 0 (0) | 0 (0) |  | 0 (0) | 2 (5.6) |  | 0 (0) | 2 (8.0) |  | 0 (0) | 1 (3.7) |  |
| Permanent cough | 0 (0) | 0 (0) |  | 0 (0) | 0 (0) |  | 0 (0) | 0 (0) |  | 0 (0) | 0 (0) |  | 0 (0) | 0 (0) |  |
| Transient  Horner’s syndrome | 1 (0.9) | 0 (0) |  | 0 (0) | 0 (0) |  | 0 (0) | 0 (0) |  | 1 (3.4) | 0 (0) |  | 0 (0) | 0 (0) |  |
| Permanent  Horner’s syndrome | 0 (0) | 2 (1.9) |  | 0 (0) | 1 (5.0) |  | 0 (0) | 1 (2.8) |  | 0 (0) | 0 (0) |  | 0 (0) | 0 (0) |  |
| Transient  Shoulder dysfunction | 0 (0) | 0 (0) |  | 0 (0) | 0 (0) |  | 0 (0) | 0 (0) |  | 0 (0) | 0 (0) |  | 0 (0) | 0 (0) |  |
| Permanent  Shoulder dysfunction | 0 (0) | 2 (1.9) |  | 0 (0) | 0 (0) |  | 0 (0) | 0 (0) |  | 0 (0) | 2 (8.0) |  | 0 (0) | 0 (0) |  |
| **Non-nerve-related complications** | 1 (0.9) | 17 (15.7) | <0.001 | 0 (0) | 4 (20.0) | 0.539 | 0 (0) | 8 (22.2) | 0.005 | 0 (0) | 3 (12.0) | 0.278 | 1 (4.3) | 2 (7.4) | 1.000 |
| Transient HP | 0 (0) | 5 (4.6) |  | 0 (0) | 2 (10.0) |  | 0 (0) | 2 (5.6) |  | 0 (0) | 0 (0) |  | 0 (0) | 1 (3.7) |  |
| Permanent HP | 0 (0) | 4 (3.7) |  | 0 (0) | 1 (5.0) |  | 0 (0) | 1 (2.8) |  | 0 (0) | 1 (4.0) |  | 0 (0) | 0 (0) |  |
| Pulmonary infection | 1 (0.9) | 6 (5.6) |  | 0 (0) | 1 (5.0) |  | 0 (0) | 3 (6.3) |  | 0 (0) | 2 (8.0) |  | 1 (4.3) | 1 (3.7) |  |
| Chyle leak | 0 (0) | 2 (1.9) |  | 0 (0) | 0 (0) |  | 0 (0) | 2 (5.6) |  | 0 (0) | 0 (0) |  | 0 (0) | 0 (0) |  |

Data are presented as n (%). P values were calculated using Fisher's exact test (two-sided). For subgroup analyses by anatomical subtype (Types I-IV), P values were adjusted for multiple comparisons using the Holm method.
